# Subacute Small Bowel Obstruction in a 50-Year-Old Woman With Complete Intestinal Malrotation: A Case Report

**DOI:** 10.7759/cureus.101712

**Published:** 2026-01-16

**Authors:** Robin Sandhu, Faiz M Hussain

**Affiliations:** 1 Department of General Surgery, Apollo Institute of Medical Sciences and Research (AIMSR), Hyderabad, IND; 2 Department of Surgery, Apollo Institute of Medical Sciences and Research (AIMSR), Hyderabad, IND

**Keywords:** adult intestinal malrotation, atypical anatomy, congenital anomaly, intestinal malrotation, intestinal obstruction, ladd’s bands, laparoscopic ladd’s procedure, midgut volvulus, secondary hypertension, small bowel obstruction

## Abstract

Intestinal malrotation is a congenital anomaly resulting from the failure of the midgut to undergo its normal 270° counterclockwise rotation during embryological development. While the vast majority of cases are seen in neonates, adult presentation is exceptionally rare and often presents with vague abdominal complaints, leading to significant diagnostic delays. A 50-year-old woman presented with diffuse, colicky abdominal pain and recurrent bilious vomiting for three days. Vital signs revealed hypertension (170/100 mmHg) and tachycardia (107 bpm). The physical examination revealed a soft, but diffusely tender, abdomen. Plain abdominal radiograph revealed dilated bowel loops consistent with small bowel obstruction. Complete blood count and serum lactate were within normal limits. Contrast-enhanced CT confirmed complete intestinal malrotation with the small bowel located on the right side and the cecum located on the left side of the abdomen. Notably, the absence of the characteristic "whirl sign" indicated symptoms were due to transient duodenal compression by Ladd's bands rather than active midgut volvulus. An exploratory laparotomy was performed, revealing atypical anatomy with an unusual Ladd's band extending from the transverse colon to the right lateral parietal peritoneum, a variation from the classic cecal origin. A Ladd's procedure (adhesiolysis) was successfully performed along with prophylactic appendectomy and cecopexy to fixate the hypermobile cecum. The patient achieved complete recovery, tolerated diet well, and was discharged on Day 6 postoperatively. Notably, the patient's de novo hypertension persisted despite successful surgical correction, prompting investigation into causes of secondary hypertension, including endocrine disorders and renovascular disease. Adult intestinal malrotation is exceptionally rare but demands careful clinical attention. This case exemplifies an unusual anatomical variation with successful surgical management, emphasizing the importance of considering malrotation in the differential diagnosis of adult small bowel obstruction. It also highlights that coexisting medical conditions like hypertension require independent evaluation and management.

## Introduction

Intestinal malrotation is a congenital anomaly resulting from the failure of the midgut's normal 270° counterclockwise rotation during embryological development [1]. This failed rotation causes the formation of fibrous stalks of peritoneal folds, known as Ladd’s bands, which form a nidus for potential obstruction [2]. The majority of the cases of malrotation are diagnosed before the first year of life and are exceptionally rare in adulthood, presenting with vague abdominal complaints, causing diagnostic delay [3]. This case is particularly unique due to de novo hypertension, episodes of resolution and exacerbation leading to differing radiological findings. This is also extremely unique due to the atypical origin of Ladd’s bands from the transverse colon instead of the cecum [2].

## Case presentation

Chief complaints

A 50-year-old woman presented to the Emergency Department with diffuse, colicky abdominal pain and associated recurrent bilious vomiting, both of which had started three days ago.

History of present illness

The patient was apparently asymptomatic three days ago, following which she experienced the sudden onset of colicky, intermittent abdominal pain. The pain aggravated with movements such as walking and was relieved with rest. The pain was associated with three to five episodes of vomiting, described as nonprojectile, bilious with food and water as content. Flatus was passed, and constipation was present. Her past surgical history was significant only for a tubectomy 20 years prior. Her past medical history was negative for any known comorbidities.

Clinical findings on examination

**Figure 1:**
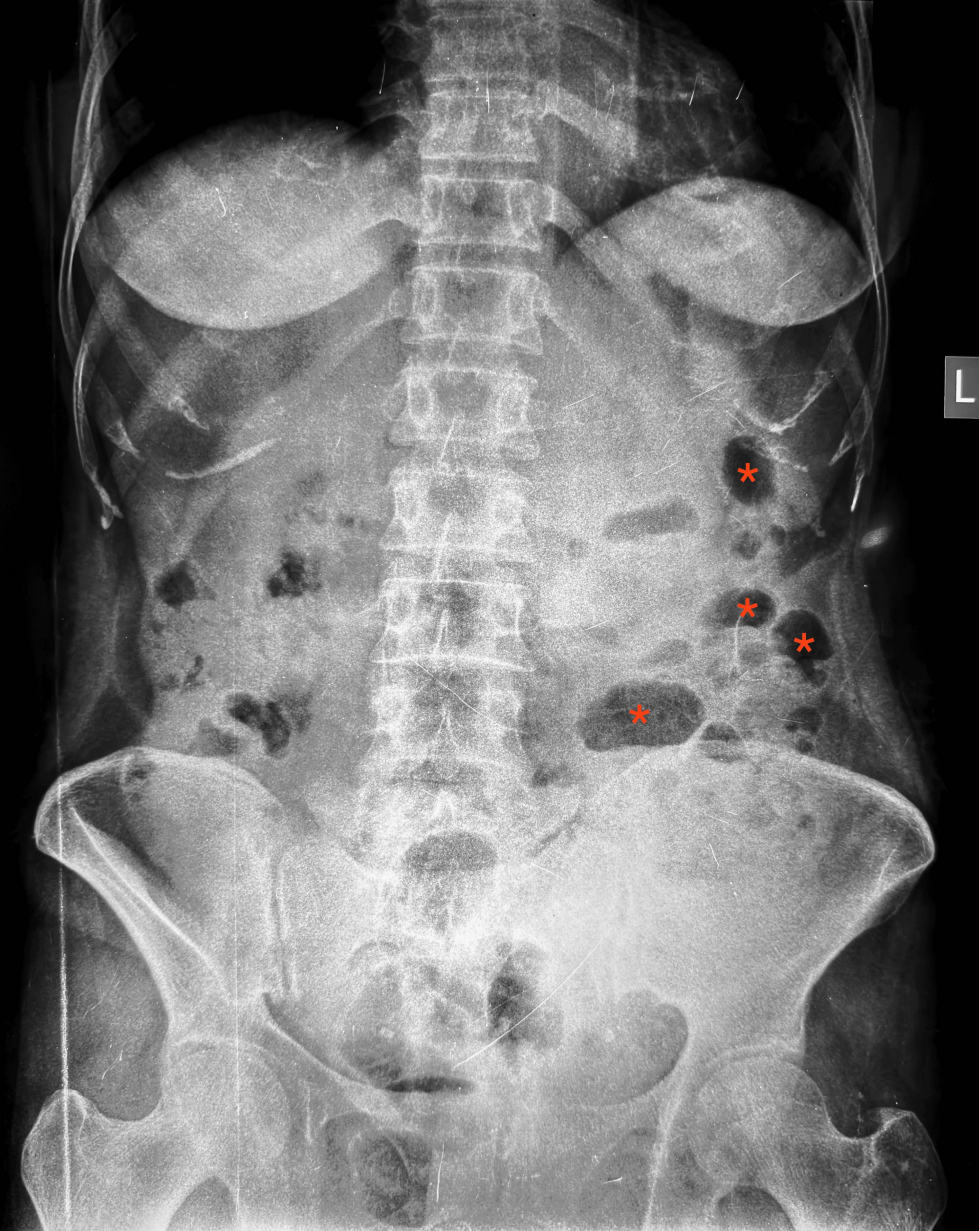
Plain abdominal radiograph showing dilated bowel loops (marked with *) consistent with obstruction

**Figure 2:**
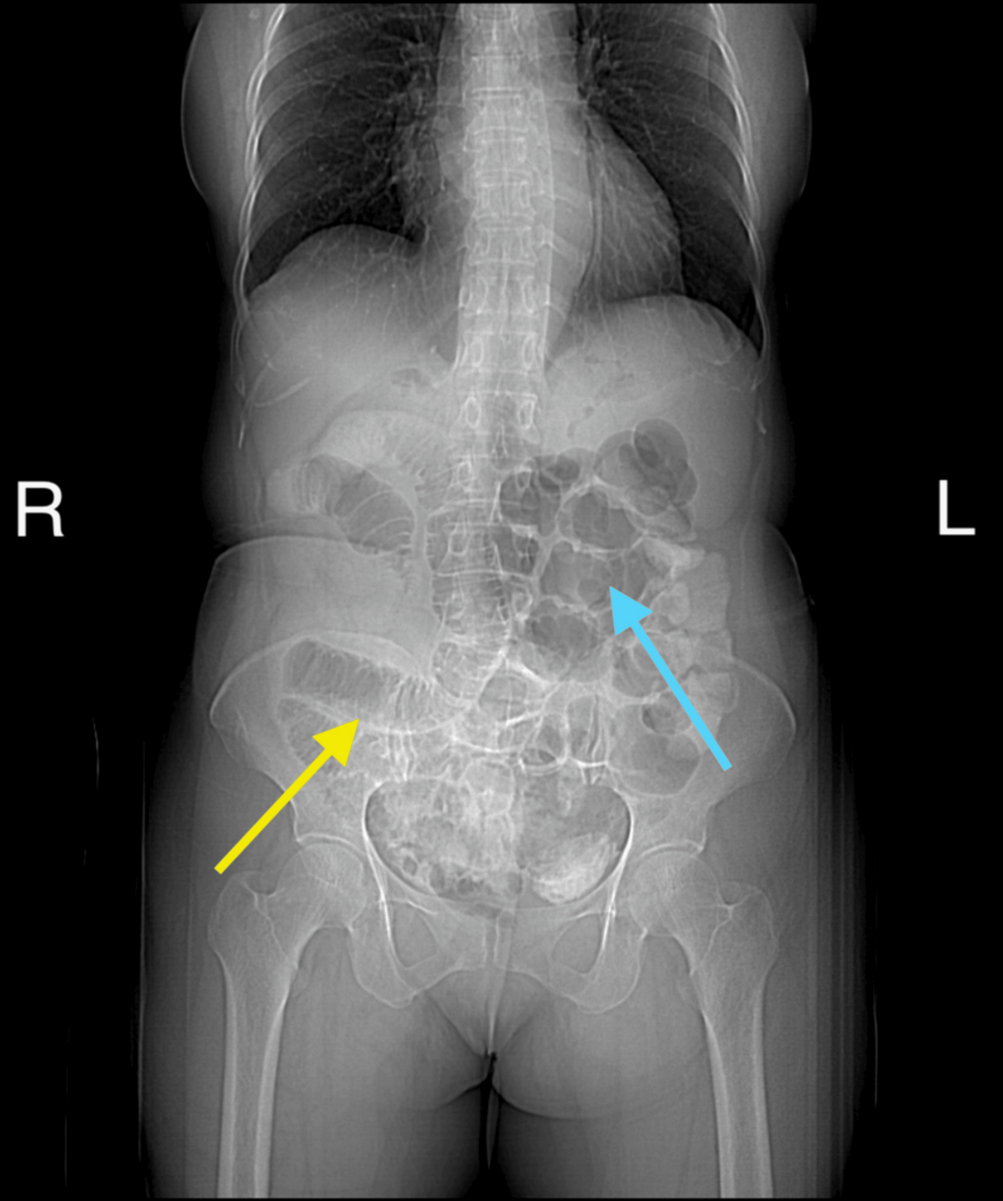
The AP view of a contrast-enhanced CT indicating the small bowel on the right side (yellow arrow) and the large bowel on the left side (blue arrow) of the abdomen AP: anteroposterior

**Figure 3:**
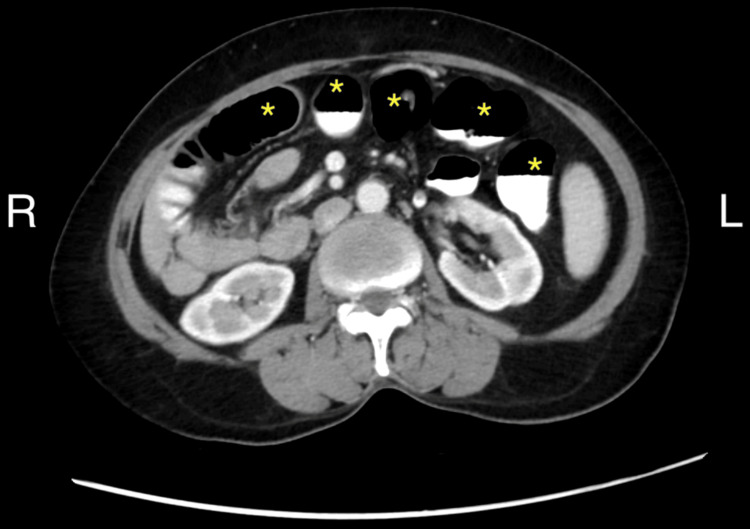
Axial contrast-enhanced CT image showing multiple fluid-filled, dilated bowel loops (marked with *) without evidence of midgut volvulus (absence of the whirl sign)

**Figure 4:**
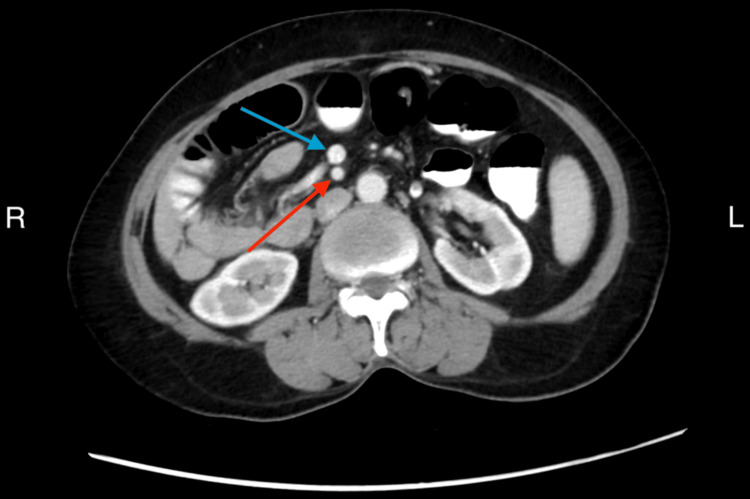
Axial portovenous phase CECT image showing the superior mesenteric artery (red arrow) and superior mesenteric vein (blue arrow) with abnormal anterior position of the vein, in keeping with intestinal malrotation CECT: contrast-enhanced CT

Upon examination, the patient was found to be hypertensive, with a blood pressure (BP) of 170/100 mmHg. She was also tachycardic (pulse 107 bpm) and tachypneic (respiratory rate 20 minutes). Her pain score was 6 out of 10. The abdominal examination was notable for a soft, diffusely tender abdomen in the right hypochondriac, right lumbar, and right iliac fossa, with audible bowel sounds. Crucially, there was no guarding or rigidity, and all quadrants moved proportionately. Per-rectal examination was normal. Blood and radiological tests were ordered, and the patient was admitted and kept nil by mouth with nasogastric decompression and urinary catheterization. The abdominal ultrasonography (USG) was negative for free peritoneal fluid, with moderate dilatation of bowel loops, as confirmed by plain radiograph (Figure 1). A working diagnosis of subacute small bowel obstruction was made, and contrast-enhanced CT (CECT) was ordered, which revealed complete intestinal malrotation with the small bowel on the right side and the large bowel on the left side of the abdomen (Figure 2), and marked dilated loops under the axial section (Figure 3). There was no “whirl sign.” The axial portovenous phase of the CECT scan demonstrated the superior mesenteric vein lying anterior to the superior mesenteric artery, an abnormal relationship consistent with intestinal malrotation (Figure 4). The patient had passed stools on the third day of admission. An arterial blood gas analysis revealed marked hypokalemia and metabolic alkalosis secondary to nasogastric losses, which were managed with infusion of potassium chloride. The complete blood count and lactate were within normal limits, indicating the absence of bowel ischemia. The absence of a whirl sign indicated the cause of the patient’s symptoms might have been due to transient compression of the duodenum by Ladd’s bands instead of mid-gut volvulus.

Given the subacute nature of obstruction, an exploratory laparotomy with prophylactic appendectomy and suspected Ladd’s band release was planned. A vertical midline incision was made. Intraoperative findings revealed the small bowel and duodenojejunal flexure on the right side, the ileocecal junction, and the large bowel on the left side. Ladd’s bands were between the transverse colon and the right lateral parietal peritoneum, noted with a hypermobile cecum. Ladd’s bands were released, along with adhesiolysis, prophylactic cecopexy, and appendectomy. The patient recovered with no residual abdominal symptoms and tolerated the diet well. The patient was started on nifedipine 10 mg orally twice daily and discharged on the sixth postoperative day.

## Discussion

The typical development of the mid-gut occurs between the fourth and twelfth week, during which the duodenum becomes retroperitoneally fixed at the ligament of Treitz, whereas the mid-gut undergoes a 270° counterclockwise rotation around the superior mesenteric pedicle [1]. Wang and Welch categorize these anomalies into subtypes, including nonrotation, malrotation, reversed rotation, and paraduodenal hernias [4]. Intraoperative findings, which reveal the small bowel on the right and the colon on the left, suggest that our patient's presentation is consistent with the nonrotation subtype. Complete or partial failure in this process results in an abnormal arrangement of the mid-gut, characterized by small bowel loops on the right side, the absence of the ligament of Treitz, and the appendix, cecum, and remainder of the colon on the left [2]. The peritoneal tissue, which normally anchors the cecum or ascending colon to the abdominal wall, is stretched and forms a fibrous stalk that passes over the duodenum to attach to the malpositioned cecum or ascending colon, creating a nidus for duodenal obstruction [2]. Almost 90% of the cases of mid-gut malrotation are diagnosed in neonates, usually within the first year of life [3]. Its persistence into adulthood is rare and often asymptomatic, and, in those who are symptomatic, it presents with vague abdominal complaints, leading to a low index of suspicion and a diagnostic challenge [1]. Symptomatic presentation in adults accounts for only about 0.2%-0.5% of overall cases [3]. Mid-gut volvulus is another complication of malrotation, where the small bowel twists around the mesentery, which, if left uncorrected, may lead to life-threatening ischemia and infarction of the involved segment [5]. Mid-gut volvulus presents with a classic whirl sign on CT and is rarely seen in adulthood, requiring early recognition and prompt management [5,6]. It may mimic other intra-abdominal pathologies; thus, cases of small-bowel obstruction in patients without predisposing factors such as previous abdominal surgeries should also raise suspicion for this entity [7]. All cases of bilious vomiting or chronic vomiting should also be properly investigated to rule out malrotation [3]. A laparoscopic Ladd’s procedure is a recommended and minimally invasive treatment that relieves duodenal compression through the separation and release of Ladd’s bands [2,8]. A consecutive prophylactic appendectomy is done as the localized symptoms of appendicitis become indistinguishable in patients with malrotation and may lead to diagnostic delays in the future [3]. This case was particularly challenging because the Ladd’s bands, which usually arise from the cecum or, less commonly, the ascending colon, originated from the transverse colon, creating an additional surgical challenge [2]. This atypical presentation challenged us as the entire vasculature, nerve distribution, and mesentery now had unconventional anatomy and unknown possibilities of complications. A precise and cautious approach is required due to the high risk of organ damage. The presence of de novo hypertension in this case also created a confounding medical emergency. Anatomical distortion from malrotation can theoretically lead to secondary hypertension through renal vessel compression. However, the persistence of hypertension in our patient after surgical correction excludes this cause. This emphasizes the need to investigate de novo hypertension independently, even in the presence of significant abdominal pathology. This also reinforces the findings of multiple studies, which have indicated that radiological testing for mid-gut malrotation via X-ray or USG may be inconclusive, and that CECT is the gold standard for diagnosis in cases of small bowel obstruction [1,5].

## Conclusions

This case highlights the diagnostic complexity of adult intestinal malrotation, a condition that is exceedingly rare in the adult population. The unusual anatomical variation observed-specifically, a Ladd's band originating from the transverse colon instead of the cecum-emphasizes the anatomical diversity that can challenge even experienced surgeons. It also underscores the importance of thorough intraoperative assessment. Importantly, this case illustrates a critical clinical principle: the persistence of hypertension following successful surgical correction of malrotation suggests that these conditions are pathophysiologically unrelated. The elevated BP (170/100 mmHg) likely indicates an independent case of secondary hypertension, necessitating a systematic investigation for underlying causes such as primary aldosteronism, renal artery stenosis, or endocrine disorders. This underscores the importance of a comprehensive medical evaluation beyond the initial surgical pathology, as it prevents premature diagnostic closure when coexisting conditions are identified. Clinicians assessing adults with recurrent, vague abdominal symptoms must maintain a heightened suspicion for congenital anomalies, regardless of the patient’s age. Prompt CECT imaging is essential for a definitive diagnosis, and a timely Ladd's procedure can prevent catastrophic complications from midgut volvulus. However, any concurrent medical conditions require independent investigation and targeted management strategies to optimize long-term patient outcomes.
